# Transcriptomic analyses of host-virus interactions during *in vitro* infection with wild-type and glycoprotein g-deficient (ΔgG) strains of ILTV in primary and continuous cell cultures

**DOI:** 10.1371/journal.pone.0311874

**Published:** 2024-10-11

**Authors:** Gayathri Gopakumar, Andrés Diaz-Méndez, Mauricio J. C. Coppo, Carol A. Hartley, Joanne M. Devlin

**Affiliations:** 1 Faculty of Science, Asia-Pacific Centre for Animal Health, Melbourne Veterinary School, The University of Melbourne, Melbourne, Victoria, Australia; 2 Escuela de Medicina Veterinaria, Universidad Andrés Bello, Concepción, Biobío, Chile; : Keele University School of Life Sciences, UNITED KINGDOM OF GREAT BRITAIN AND NORTHERN IRELAND

## Abstract

Infectious laryngotracheitis (ILT) remains a significant concern for the poultry industry worldwide due to its impact on animal welfare and its substantial economic consequences. The disease is caused by the alphaherpesvirus, *infectious laryngotracheitis virus* (ILTV). This study investigated *in vitro* host-virus interactions of a glycoprotein G (gG) deletion mutant vaccine strain of ILTV (ΔgG ILTV), and its parent wild-type strain (CSW-1 ILTV). Inoculations were performed separately for the two strains of ILTV using both a primary (chicken embryonic kidney, CEK) and a continuous culture (leghorn male hepatoma, LMH) of chicken cells. Transcriptome analysis was performed at 12 hours post infection. Each cell-type displayed distinct effects on host and viral gene transcription, with a greater number of viral and host genes differentially transcribed in CEK cells and LMH cells, respectively. Both cell-types infected with either strain demonstrated enrichment of pathways related to signalling, and gene ontologies (GO) associated with chemotaxis. Infection with either strain upregulated both SOCS proteins and certain proto-oncogenes, which may contribute to prolonged viral persistence by promoting immunosuppression and preventing apoptosis, respectively. Patterns of gene transcription related to cytokines, chemokines, endosomal TLRs, and interferon responses, as well as pathways associated with histone acetylation, transport, and extracellular matrix organization were similar within each cell type, regardless of the viral strain. In CEK cells, GO terms and pathways were downregulated uniquely after CSW-1 ILTV infection, indicating a viral-strain specific effect in this cell-type. Overall, this study highlights that the observed differences in host and ILTV gene transcription *in vitro* were more strongly influenced by the cell-types used rather than the presence or absence of gG. This underscores the importance of cell-line selection in studying host-virus interactions and interpreting experimental results.

## Introduction

Infectious laryngotracheitis (ILT) is an avian respiratory disease of economic significance caused by *Iltovirus gallidalpha 1* [1], also known as infectious laryngotracheitis virus (ILTV) [2]. The double stranded DNA genome of ILTV is approximately 150 kbp in length and encodes 80 open reading frames (ORFs), with the majority of these, homologous to the ORFs of herpes simplex virus type 1 (HSV-1) [3]. An earlier study classified the genes of ILTV into four categories based on their transcription patterns as, α, β, γ 1 and γ 2 genes, similar to that of HSV-1 [4], while a more recent study has classified them into immediate-early (IE), early (E), early late (E/L) and late (L) genes, in recognition of the genes that shared features of both early and late genes [5].

The virus is transmitted horizontally through the respiratory or ocular routes [6–8] and undergoes replication in the mucosal epithelial cells causing pathology and disease [9–11]. The outcome of ILTV infection depends on the virulence of the strain, viral load, age of infected birds, vaccination status, as well as synchronous infection with other respiratory pathogens [2,12–15]. Following infection, the virus establishes life-long latency in trigeminal ganglia and tracheal tissues and can reactivate during periods of stress [9,16].

Devlin *et al*. identified glycoprotein g (gG) of ILTV as a virulence factor [17,18]. A gG deletion mutant live attenuated strain of the virus (ΔgG ILTV) was therefore proposed and used as a vaccine candidate against ILT [17,19]. Subsequent studies also determined that ILTV gG is a viral chemokine binding protein (vCKBP) [20], capable of influencing the expression of several other ILTV genes [21], as well as a selected set of host chemokine and cytokine genes *in vitro* and *in vivo* [22]. Although the safety and efficacy of this vaccine have been demonstrated in several previous studies [18,19,23,24], the underlying molecular mechanism and signalling pathways involved during infection with this vaccine (ΔgG ILTV) or the parent wild-type strain (CSW-1 ILTV) remains incompletely characterised, prompting global gene expression studies of these ILTV strains and the host, in parallel.

Genome-wide gene expression studies of host response to wild-type and vaccine strains of ILTV have utilised both *in vitro* and *in vivo* systems [25–27], each having their own advantages and disadvantages. While primary cultures of chicken embryo lung, chicken embryo liver and chicken embryo kidney cells (CEK) are more sensitive to ILTV infection, the use of the only ILTV permissive commercial cell line, the chicken Leghorn male hepatoma cell line (LMH; ATCC CRL- 2117) [28], offers more practicality [29]. Being a continuous cell line, LMH cells are also devoid of the inherent heterogeneity associated with primary cell cultures [29], and several studies in the recent past have demonstrated its use for the study of metabolic requirements of ILTV and host factors that influence ILTV replication and spread [30–33].

This study aimed to evaluate cell-type specific and virus-strain specific differences during ILTV infection *in vitro*, using RNA-seq analysis. To understand ILTV-host interactions across different cell culture systems, a primary culture of CEK cells and the continuous LMH chicken cell line were used. To further examine the role of gG during *in vitro* infection, the gG deletion mutant vaccine strain ΔgG ILTV and the gG- expressing parent wild type CSW-1 ILTV strains were used.

## Materials and methods

### Ethics statement

The isolation of primary chicken embryo kidney (CEK) cells was approved and performed in accordance with the ethical guidelines of the Animal Ethics Committee Faculty of Veterinary and Agricultural Science (FVAS), The University of Melbourne (ethics identifier: 1814492.2).

### Cells

Leghorn male hepatoma cell cultures (LMH; ATCC CRL- 2117) [28] were maintained in low glucose Dulbecco’s modified Eagle’s medium (DMEM), supplemented with 5% v/v foetal bovine serum (FBS, Sigma-Aldrich Cat. No. 12003C-500ML, SAFC Industries, Buchs, Switzerland), 100 μg/mL ampicillin, 100 μg/mL gentamicin and 10 mM 2-hydroxyethyl-1-piperazineethanesulfonic acid (HEPES [pH 7.7]). Chicken embryo kidney cells were harvested from eighteen-day-old specific pathogen-free (SPF) chicken embryos (Australian SPF services). Following the ‘AVMA guidelines for the euthanasia of animals: 2020 edition’, embryos were decapitated immediately after they were removed from the egg shell and CEK cells were isolated by enzymatic digestion as described previously [34]. These cells were maintained in CEK growth media (low glucose DMEM, supplemented with 5% v/v FBS, 5 μg/mL amphotericin B) in tissue culture flasks or plates pre-coated with 0.1% w/v gelatine and incubated at 37°C in 5% v/v CO_2_ in humidified air in a cell culture incubator until use.

### Viral strains

This study used CSW-1 ILTV (an Australian virulent field strain of ILTV), and also a gG-deficient ILTV strain (ΔgG ILTV) that was generated from the parent wild-type CSW-1 ILTV strain by homologous recombination [18]. Viral strains sourced from laboratory stocks were further propagated once each in CEK cells and twice each in LMH cells. They were then subjected to ultra-centrifugation at 50,000 *× g* for 1 hour at 4°C and resuspended in low glucose DMEM, supplemented with 5% v/v FBS, to generate high titre virus stocks. Viral stocks were then titrated by plaque-forming unit assays on LMH cells [18].

### ILTV infection and sampling

LMH and CEK cell monolayers, grown to approximately 90% confluency in 6-well tissue culture plates, were employed for virus inoculation. Six wells each of these cells were either mock-inoculated with sterile medium (DMEM supplemented with 5% v/v FBS) or inoculated with the CSW-1 or the ΔgG strains of ILTV at a multiplicity of infection (MOI) of 3.5. The inoculated plates were incubated for 1 hour at 37°C, with rocking every 10 minutes to allow viral adsorption. Following incubation, inocula were removed and cells were washed three times with 2 mL of phosphate-buffered saline (PBS) containing antimicrobials (100 μg/mL ampicillin, 5 μg/mL amphotericin B) before 2 mL of fresh growth medium appropriate for each cell type was added to the wells. The plates were gently swirled and then 500 μL of medium was immediately collected from each well and stored at -80°C to be used as the initial time point reference at T = 0 hours post infection (hpi) for ILTV detection and quantification in the cell culture supernatant using a qPCR assay. The plates were then incubated at 37°C in 5% v/v CO_2_ in humidified air in a cell culture incubator. At twelve- hpi, cell culture supernatants were collected from triplicate wells and stored at -80°C prior to their use for the detection and quantification of ILTV DNA by qPCR. The cell monolayers were washed once with PBS, flooded with 600 μL RLT buffer (RNeasy Mini Kit, QIAGEN) containing 1% (v/v) ß-mercaptoethanol, collected and stored at -80°C until RNA extractions were performed.

### DNA extraction

DNA was extracted from 200 μL each of the supernatant samples using MagMAX CORE Nucleic Acid Purification Kit (Applied Biosystems) with the aid of the KingFisher Flex Purification System (Thermo Scientific) automated DNA extraction platform in 96-well plates. From each sample 90 μL of the eluate was collected and stored at -80°C until the qPCR assay was conducted.

### Detection of ILTV DNA in supernatant samples using ILTV UL15 qPCR

DNA samples were subjected to qPCR for the determination of genome copy number (GCN). The protocol developed by Mahmoudian *et al*. and modified by Thilakarathne *et al*. was adopted, targeting the ILTV UL15 gene [35,36]. DNA amplification standard curves were generated using the QIAgility Robot (QIAGEN) that prepared 10-fold serial dilutions of the pGEM-T (Promega) plasmid containing the 113 bp fragment of the ILTV UL15 gene. Standards were run in triplicate while the test samples in singlicate. The qPCR reaction and data acquisition were performed using the Rotor-Gene Q thermocycler (QIAGEN). One hundred copies per reaction were used as the cut-off for detection and quantification of ILTV DNA.

### RNA extraction

Monolayers of LMH and CEK cells collected at 12 hpi were subjected to RNA extraction as described in a previous study [37]. Briefly, total RNA extracted using RNeasy Mini kit (QIAGEN) following manufacturer’s instructions were subjected to DNase treatment using the Turbo DNA-free kit (Invitrogen) and then cleaned and concentrated using Zymo RNA clean and concentrator-25 (Zymo Research Corporation) according to manufacturers’ instructions. RNA eluates were immediately stored at -80°C. The quality of total RNA was assessed using the Agilent 4200 TapeStation system (Agilent Technologies) and all RNA samples were verified to have an RNA integrity number (RIN) > 8 prior to complementary DNA (cDNA) library preparations.

### cDNA library preparation and Illumina sequencing

Methodology used for cDNA library preparation and Illumina sequencing has been described in a previous study [37]. Briefly, using the TrueSeq Stranded mRNA library preparation kit (Illumina Inc.), poly-A mRNA was isolated from 400 ng of total RNA from each replicate, using poly-T-oligo magnetic beads, as per the manufacturer’s recommendations. Purified mRNA was then fragmented, and reverse transcribed to produce first strand cDNA using random primers and PCR amplification. This was followed by second strand cDNA synthesis using DNA polymerase I and RNase H. Prime ends of cDNA were then adenylated and subjected to single index adaptor ligation. The end products were purified and enriched by PCR for the generation of the final cDNA libraries. The quality of the cDNA libraries was assessed using the Agilent 4200 TapeStation system (Agilent Technologies) and all samples conformed to the required fragment size (approximately 260 bp) for sequencing. Libraries were then normalized to 1nM, pooled, denatured and sequenced on a NextSeq500 Sequencing platform (Illumina Inc.) using the paired-end approach.

### RNA-seq data analysis

Bioinformatic analyses of RNA-seq data were conducted on the bioinformatics web-based- analysis and workflow platform ‘GALAXY’ [38] at usegalaxy.au, following the Galaxy workflow [39,40]. Quality assessment of the raw reads was performed using FastQC (Galaxy Version 0.72+galaxy1). Reads in pairs were subjected to trimming using Cutadapt (Galaxy Version 3.4+galaxy1) and bases with PHRED quality score > 20 and reads > 20 bp in length were selected for downstream analyses. Ensemble database release 104 of the annotated chicken (*Gallus gallus*) genome (gene transfer format [gtf] and FASTA format) and the CSW-1 ILTV genome, GenBank accession number: JX646899.1 (gene transfer format [gtf] and FASTA format), were used as reference sequences to perform read mapping to the chicken genome and to the ILTV genome, respectively. Mapping was performed using the alignment program ‘hierarchical indexing for spliced alignment of transcripts’ (HISAT2, Galaxy version 2.2.1+galaxy0) [41]. Read summarisation on BAM files outputs were performed using featureCounts (Galaxy Version 2.0.1+galaxy1) to generate exon-level read counts. Fragments (pairs of reads) with a minimum mapping quality score (minMQS) of less than 10, or reads that aligned to multiple or overlapping features, were exclude from the read-counts files of chicken genes. However, to enable read counting in the repeat regions of ILTV genome, multi-mapped reads were included during read summarisation of the ILTV genes.

### Differential gene expression analysis

Differential gene expression analysis was carried out using DEseq2 [42] in Galaxy (Galaxy Version 2.11.407 +galaxy2). Protein-coding host genes or open reading frames (ORFs) of ILTV with Padj value (p-value corrected for multiple testing using the Benjamini-Hochberg procedure for the Wald statistic) < 0.01 and with ≥ 2-fold change in expression levels in the CSW-1, or the ΔgG ILTV group, as compared to the respective Mock-inoculated group in each cell type (four comparisons in total) were considered as significantly differentially regulated.

### Gene Ontology (GO), pathway and protein class analysis

Genes up- or down-regulated significantly after ILTV inoculation (compared to mock) were subjected separately to gene ontology (GO), pathway and protein class analyses using the Panther (version 17.0) data base [43]. GO terms, Reactome pathways and protein classes enriched with the upregulated and downregulated host genes for the 4 comparisons, at P value < 0.05 were considered significant. GO terms enriched for categories of biological process (BP), cellular component (CC) and molecular function (MF) were further summarised using the web server REVIGO [44] and reported.

### Statistical analyses and data visualisation

Statistical analyses of the normally distributed qPCR data (determined by the Shapiro Wilks test) were carried out using Welch’s t-test corrected for standard deviations on Prism-GraphPad Version 9.4.1. A p-value lower than 0.05 was considered statistically significant. Data visualisation was performed using Prism GraphPad Version 9.4.1, R Studio (2022.07.2), R version 4.0 and SRplot.

## Results

### 1. Active replication of CSW-1 ILTV and ΔgG ILTV in LMH and CEK cells

**Fig 1:**
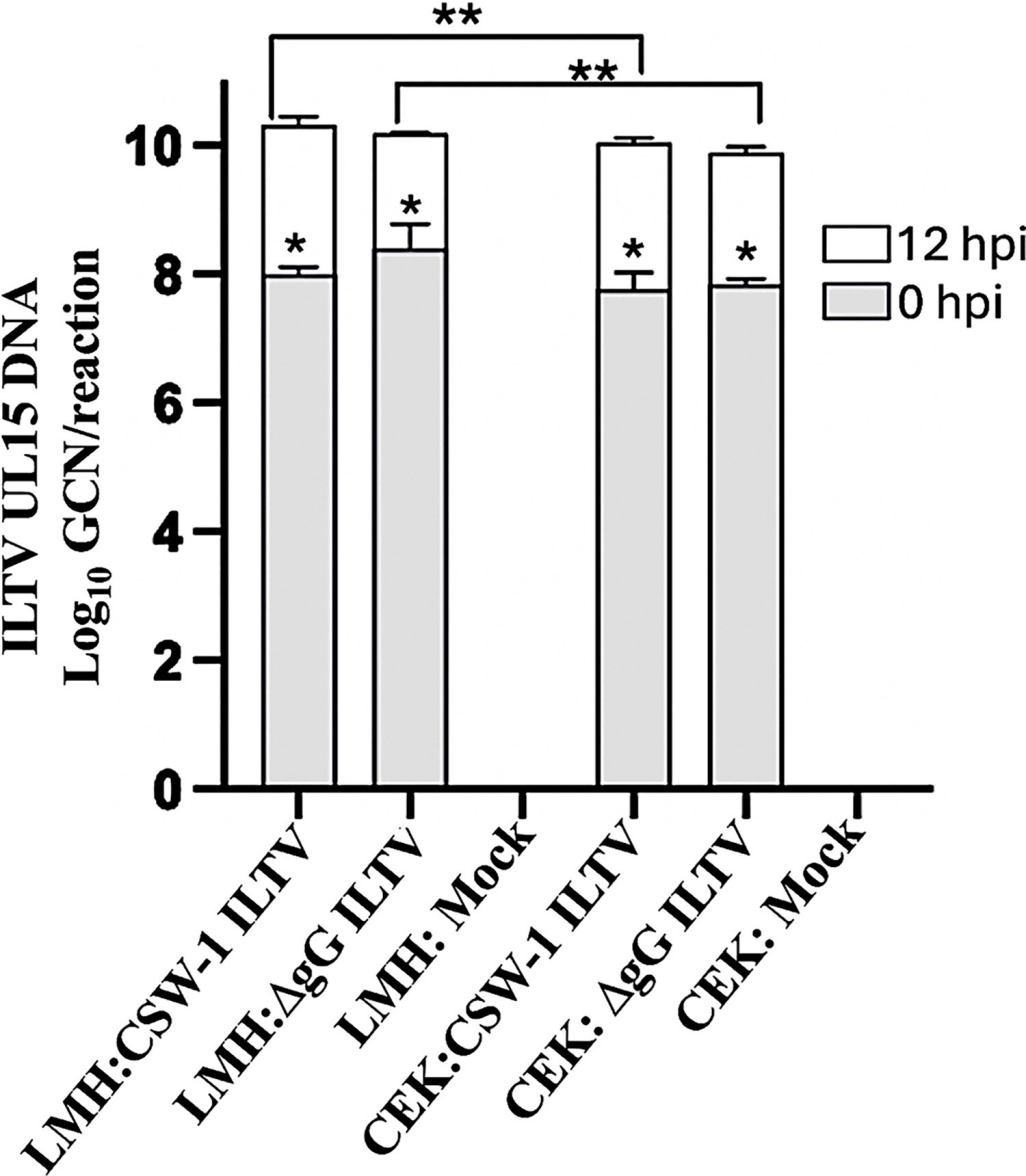
ILTV genome copy numbers in cell culture supernatant. Stacked bar plot representing log_10_ genome copy numbers per reaction of ILTV UL15 DNA in the supernatant samples collected after mock inoculation of LMH and CEK cells with CSW-1 ILTV or ΔgG ILTV at an MOI of 3.5. Genome copy number of samples collected at 0hpi is indicated by shaded bars and at 12hpi is indicated by clear bars. Error bars indicate mean standard deviation. P-value < 0.05 was considered statistically significant. Asterisks within the bars indicate significant differences between time points within each infection group. Asterisks across bars indicates significant differences across the same virus strain in different cell types. *p- value ≤ 0.014, ** p- value ≤ 0.041.

With a limit of detection of 100 copies per reaction, the ILTV UL15 qPCR detected DNA in LMH and CEK cells inoculated with CSW-1 ILTV or ΔgG ILTV, but not with sterile media (mock). A significant increase in ILTV GCN from 0 hpi to 12 hpi with CSW-1 ILTV and ΔgG ILTV in LMH (*P* < 0.0001 and = 0.014, respectively) and CEK cells (*P* = 0.0023 and < 0.0001, respectively) indicated active replication of the viral strains and the progress of infection over time in both cell-types (Fig 1). No significant differences in ILTV GCN levels were observed between samples of the CSW-1 ILTV and the ΔgG ILTV inoculated groups at 0 hpi or at 12 hpi, within each cell type (S1 Table). However, the genome copy numbers were significantly different between the groups inoculated with the same strain, across the two cell-types at 12 hpi (*P =* 0.0417 in LMH cells and 0.0250 in CEK cells).

### 2. RNA-seq analysis

Pre-processed and trimmed RNA-seq reads were mapped separately to the reference chicken genome and to the CSW-1 ILTV genome (S2 Table**)**. The principal component analysis (PCA) plots of DESeq2 differential gene expression analyses displayed clustering of biological replicates with clear distinction of groups (S1A and S2A Figs). With the differentially expressed host genes, groups were separated largely (98% variance) on principal component (PC) 1. The sample-to-sample distance plot indicated that the difference in host gene expression between the groups were predominantly based on difference in cell-type **(**S1B Fig). The PCA plot of the differentially expressed viral genes indicated separation of groups based on cell-type (PC1 69% variance) and the viral strain (PC2 21% variance). The sample-to-sample distance map highlighted clustering of replicates that were first grouped by cell-type and then by the type of viral strain used (S2B Fig).

### 3.Differentially expressed genes (DEGs)

#### i) Differentially expressed host genes

Differential gene expression analysis with a criterion of Padj value (P-value corrected by Benjamin Hochberg method) < 0.01, with greater than or equal to 2-fold change **(**log_2_FC 1) in expression levels identified the host genes that were differentially expressed in the ILTV-inoculated groups as compared to the mock-inoculated groups in each cell type. A larger number of genes was upregulated than downregulated in all the comparisons (Table 1 and S14–S21 Tables). Infection of LMH cells (as compared to CEK cells) and infection with CSW-1 ILTV (as compared to ΔgG ILTV) resulted in the differential regulation of a larger number of host genes, reflecting cell-specific and viral strain-specific differences in overall differential gene expression. The top 10 annotated genes that were up- or downregulated following infection with CSW-1 ILTV or ΔgG ILTV in each cell type are shown in S3–S6 Tables in supporting information. This included genes that encoded proteins with functions related to signalling, transcriptional regulation, immune response, metabolism, cell adhesion and proliferation, transport as well as the maintenance of cytoskeleton.

**Table 1:** Differentially expressed host genes in LMH or CEK cells at 12 hours post inoculation with CSW-1 ILTV or ΔgG ILTV compared to the respective mock-inoculated cell type.

Cell type	Comparison	Total number of Sig DEG*	Sig Up^†^	Sig Down^¶^
**LMH**	CSW-1 ILTV vs Mock	2443	1287	1156
	ΔgG ILTV vs Mock	876	482	394
**CEK**	CSW-1 ILTV vs Mock	1581	1026	555
	ΔgG ILTV vs Mock	521	473	48

*Significantly differentially expressed genes

^†^Genes significantly upregulated at Padj < 0.01and log_2_FC ≥ 1

^¶^Genes significantly downregulated at Padj < 0.01and log_2_FC ≤ -1.

**Fig 2:**
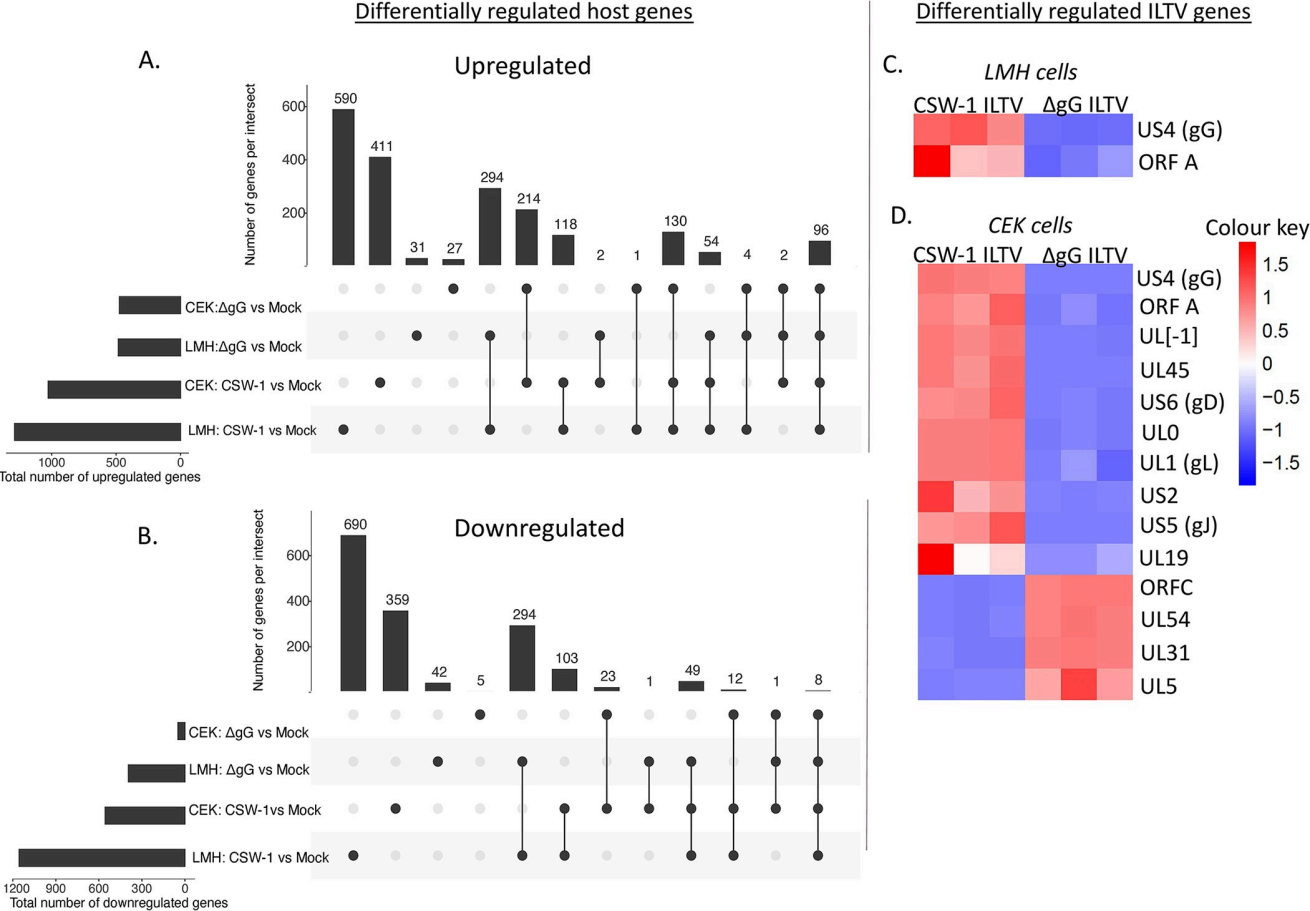
Differentially regulated host and ILTV genes. A) and B) show UpSetR plots representing the sets of common and unique host genes that were A) upregulated (Padj < 0.01, log_2_ FC ≥ 1) or B) downregulated (Padj < 0.01, log_2_ FC ≤ -1) in CEK or LMH cells at 12 hours post-inoculation with CSW-1 ILTV or ΔgG ILTV (compared to mock). The horizontal bar chart on the left of (A and B) indicates the total number of host genes differentially regulated after infection in each of the systems. The vertical bar chart on the top indicates the intersection size between sets of host genes differentially regulated in one or between multiple comparisons. Black dots underneath each bar shows the culture system (left) represented by the bar above. Black dots connected by vertical lines indicate the culture systems of the comparison, where the bar above shows the number of genes in common. Heatmaps representing the ILTV genes differentially regulated between CSW-1 ILTV and ΔgG ILTV are shown in C) LMH cells and D) CEK cells, respectively. Rows represent genes and columns represent samples. Gene expression levels are represented by normalised read counts for triplicates and are reflected by the intensity of colour in the squares shared by the gene and the corresponding sample; red colour indicates upregulation (Padj < 0.01, log_2_ FC ≥ 1) and blue colour indicates downregulation (Padj < 0.01, log_2_ FC ≤ -1).

A large proportion of the up or downregulated genes were shared commonly following infection with either strain of ILTV in both cell-types (Fig 2A and 2B respectively). A total of 23% (294/1287) and 61% (294/482) of the upregulated genes (Fig 2A), and 25% (294/1156) and 75% (294/394) of the downregulated genes (Fig 2B) were shared between LMH cells inoculated CSW-1 ILTV and ΔgG ILTV, respectively. A total of 23% (214/1026) and 45% (214/473) of the upregulated genes (Fig 2A), and 4% (23/555) and 21% (23/48) of the downregulated genes (Fig 2B) were shared between CEK cells inoculated CSW-1 ILTV and ΔgG ILTV, respectively. The majority of the upregulated cytokines and chemokines were similar within each cell type (Table 2). The gene encoding IL-8 was the most upregulated chemokine/cytokine encoding gene in LMH cells, while those encoding IL-17C and IL-19 were most upregulated in CEK cells inoculated CSW-1 ILTV and ΔgG ILTV, respectively. Interestingly, the expression of IL-12B and IL2RG was upregulated in both LMH and CEK cells infected with CSW-1 ILTV, but not with ΔgG ILTV. Differentially regulated interferon-related genes included a few up- and downregulated genes in LMH cells, but not in CEK cells (Tables 2 and 3). The only downregulated chemokine/cytokine encoding genes were IL-15 and LECT2 that were downregulated in LMH cells and CEK cells inoculated with CSW-1 ILTV and ΔgG ILTV, respectively (Table 3). In addition to this, the expression of several receptors for interleukins, and several endosomal toll-like receptors were uniquely downregulated in LMH cells (Table 3).

**Table 2:** Host genes with immune-related functions upregulated in LMH and CEK cells inoculated with CSW-1 ILTV or ΔgG ILTV at 12 hours post inoculation.

Gene ID	Log_2_ Fold change			
	LMH CSW-1 ILTV	LMH ΔgG ILTV	CEK CSW-1 ILTV	CEK ΔgG ILTV
*Cytokines*, *Chemokines and receptors*				
IL-8	7.46	5.09	2.91	2.75
IL-8L1	6.76	4.54	3.3	2.73
IL-4I1	5.28	3.91	3.31	2.19
IL-12B	4.60	N	1.90	N
IL-17REL	3.44	2.79	N	N
IL-1B	3.39	N	N	N
IL-2RG	2.82	N	1.32	N
IL-1RL1	2.24	2.08	1.50	N
IL-11	2.19	N	1.90	1.32
IL-7R	1.32	1.15	N	N
IL-17RD	1.25	1.17	N	N
IL-17C	N	N	3.52	N
IL-19	N	N	3.10	3.11
IL-10RA	N	N	3.00	1.9
IL-1R2	N	N	2.25	2.19
IL-6	N	N	2.08	1.23
CXCR-4	2.93	N	2.57	1.71
CCR-7	1.29	1.26	N	N
CCL-4	1.20	1.05	3.28	1.60
CXCR-5	N	N	3.35	2.47
CCL-20	N	N	3.13	2.30
CXCL-13L2	N	N	2.56	N
CCL-17	N	N	2.13	1.84
CXCL-13L3	N	N	1.71	N
CXC-R1	N	N	1.67	N
CXCL-14	N	N	1.66	N
*Interferon- related genes*				
IRF1	3.89	1.74	N	N
IFRD1	2.37	N	N	N
IFITM5	1.17	N	N	N

Padj value < 0.01and log_2_FC ≥1 was considered significantly upregulated, N; not upregulated.

**Table 3:** Host genes with immune-related functions downregulated in LMH and CEK cells inoculated with CSW-1 ILTV or ΔgG ILTV at 12 hours post inoculation.

Gene ID	Log_2_ Fold change			
	LMH CSW-1 ILTV	LMH ΔgG ILTV	CEK CSW-1 ILTV	CEK ΔgG ILTV
*Cytokines*, *Chemokines*, *and receptors*				
IL15	-2.81	N	N	N
IL20RA	-2.36	N	N	N
IL2RB	-1.56	N	N	N
IL1RL2	-1.48	N	N	N
IL11RA	-1.32	N	N	N
IL22RA1	N	N	-1.88	N
IL1R1	-1.13	N	N	N
IL13RA1	-1.31	N	N	N
LECT2	N	N	N	-1.37
*Endosomal toll-like receptors*				
TLR7	-2.70	-2.28	N	N
TLR21	-1.20	N	N	N
TLR3	-1.06	N	N	N
*Interferon-related genes*				
IRF6	-1.07	N	N	N
IRF2	-1.05	N	N	N

Padj value < 0.01and log_2_FC ≤- 1 was considered significantly downregulated, N; not downregulated.

#### ii) Differentially expressed viral genes

Using the same criteria as was used for differential host gene expression analysis, ILTV genes that exhibited significant differences in expression in the CSW-1 ILTV inoculated group as compared to the ΔgG ILTV inoculated group in each cell type were identified (Fig 2C and 2D). Apart from the late gene ORF71 (US4) that encodes gG, the expression of another late gene, ORF13 (ORFA), was significantly upregulated in the CSW-1 ILTV inoculated groups in both cell-types. In addition to this, the expression of several other ILTV genes were differentially regulated between the two strains in CEK cells but not in LMH cells. Genes for which the expression levels were significantly upregulated in the CSW-1 ILTV inoculated CEK cells included the late genes [5] ORF12 (UL45), ORF63 (UL0), ORF 64 (UL[–1]), US5 (glycoprotein J, gJ) and ORF68 (US2), the early genes ORF44 (UL19) and UL1 (glycoprotein L; gL), and an early/late (E/L) gene US6, glycoprotein D. Genes for which the expression levels were significantly downregulated in the CSW-1 ILTV inoculated CEKs cells included the early genes ORF3 (UL54), ORF57 (UL5) and ORF28 (UL31) as well as the late gene ORF15 (ORFC).

### 4. Gene ontology analysis

**Fig 3:**
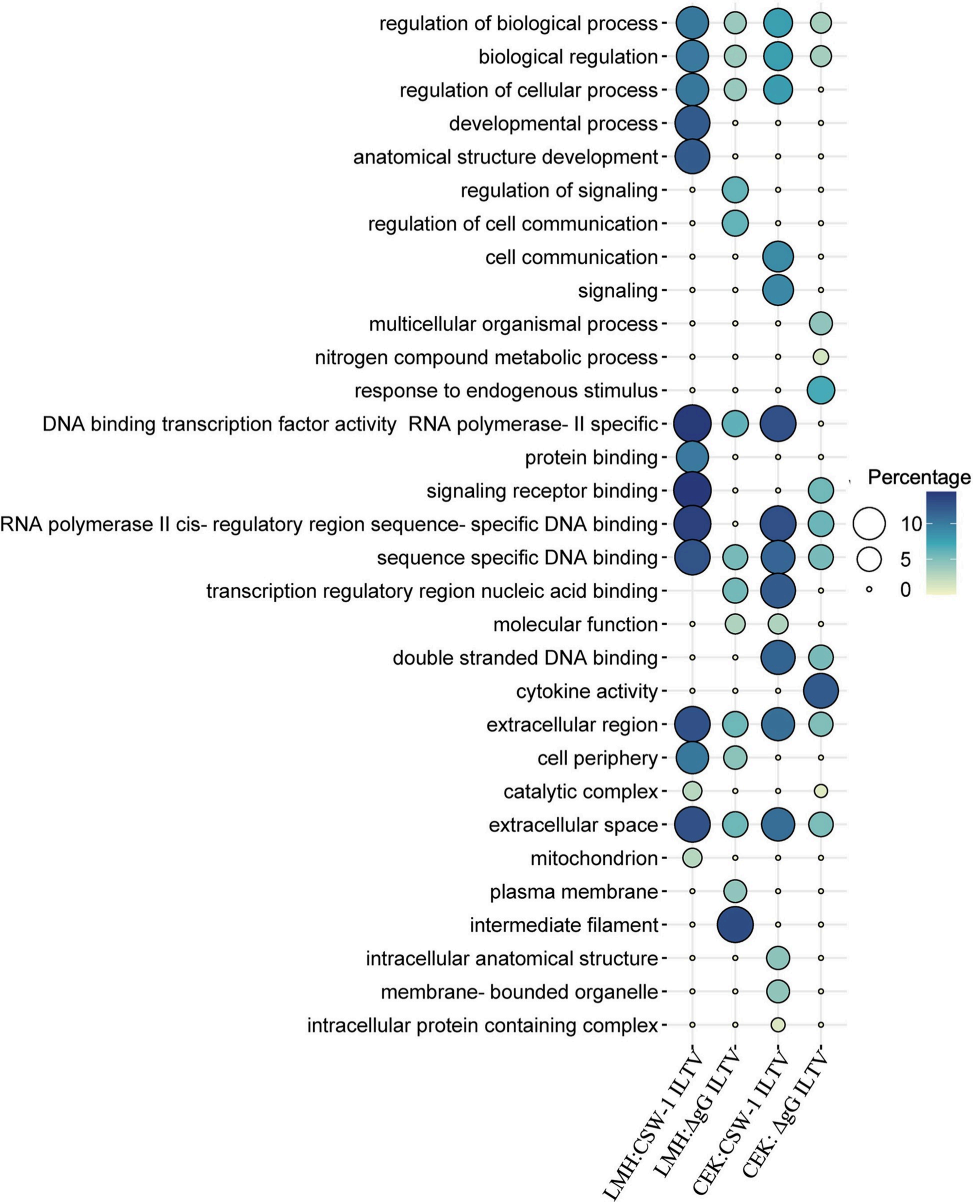
Top 5 upregulated gene ontology (GO) terms. GO terms for biological processes, molecular functions and cellular components enriched with the genes upregulated (Padj <0.01, log_2_ FC ≥ 1) in LMH and CEK cells, inoculated with CSW-1 ILTV or ΔgG ILTV at 12 hours post inoculation are represented as bubble plots. The size and colour of bubbles are proportional to the percentage of genes enriched for the GO term.

GO analysis using Panther identified the BPs, MFs and CCs enriched with the up- or downregulated genes at FDR < 0.05, that were further summarised using REVIGO (S7–S13 Tables). Many of the upregulated GO terms were similar across all comparisons. The top 5 category (Fig 3) of upregulated GOs included the BP terms that were mainly related to the regulation of biological and cellular processes and processes associated with signalling. Developmental processes were enriched uniquely in LMH cells, while processes related to metabolism and response to stimulus were enriched uniquely in CEK cells (Fig 3). The MF terms indicating DNA binding were enriched across all 4 comparisons, while functions related to protein binding and cytokine activity were enriched uniquely in LMH cells infected with CSW-1 ILTV and in CEK cells inoculated with ΔgG ILTV respectively (Fig 3). The CC terms for extracellular region and extracellular space were enriched commonly in all 4 comparisons, while cell periphery was highlighted uniquely in LMH cells inoculated with either strain of ILTV (Fig 3).

**Fig 4:**
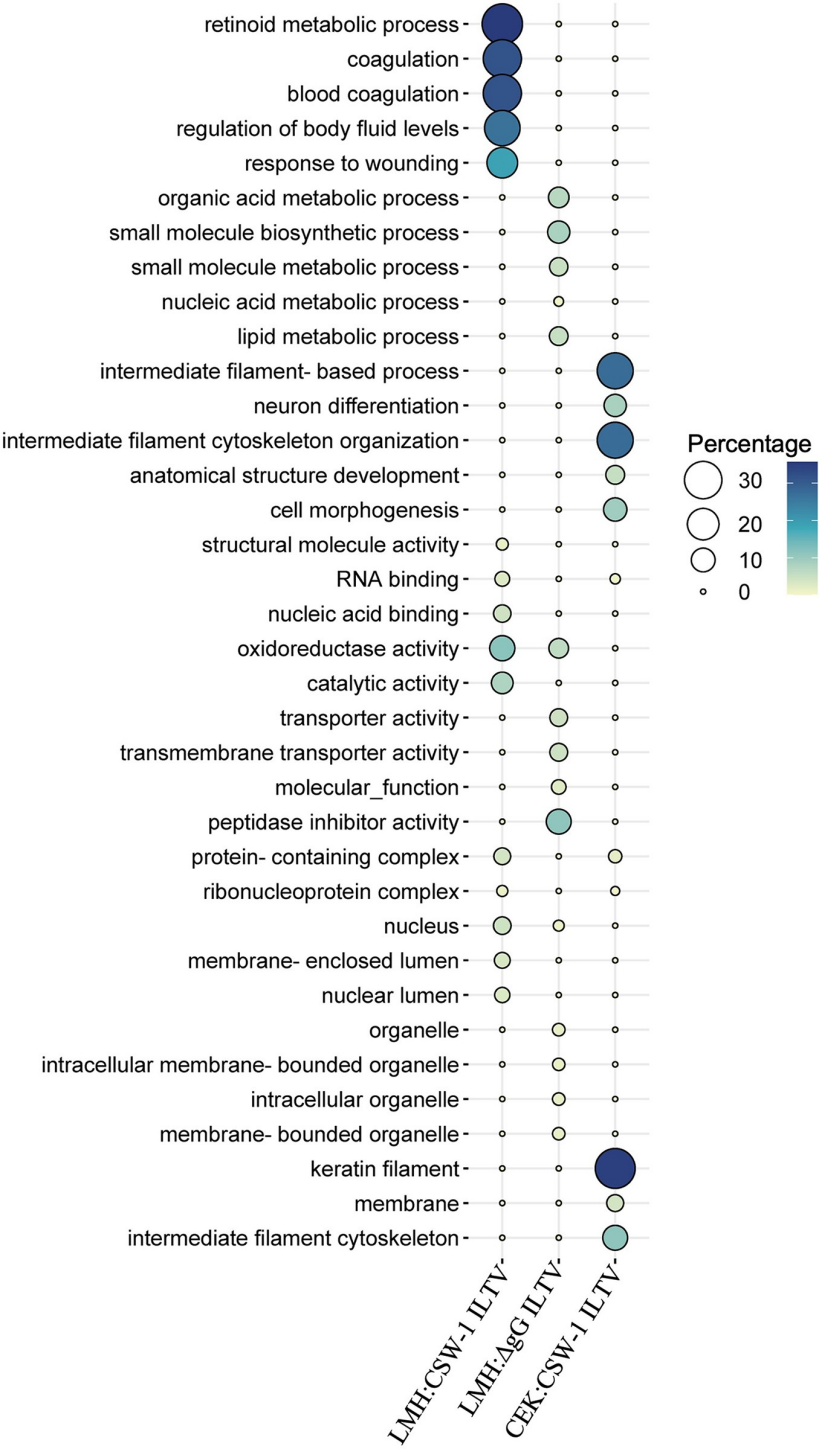
Top 5 downregulated gene ontology (GO) terms. GO terms for biological processes, molecular functions and cellular components enriched with the genes downregulated (Padj < 0.01, log_2_ FC ≤ - 1) in LMH and CEK cells, inoculated with CSW-1 ILTV or ΔgG ILTV at 12 hours post inoculation are represented as bubble plots. The size and colour of bubbles are proportional to the percentage of genes enriched for the GO term.

No GO terms were enriched with the genes that were downregulated in CEK cells inoculated with ΔgG ILTV. The top 5 category of downregulated BPs (Fig 4) included terms related to metabolic processes, that were enriched in LMH cells inoculated with either strain of ILTV. This also included terms related to wounding and blood coagulation in LMH cells inoculated with CSW-1 ILTV and terms related to cytoskeletal and structural organisation, cell development and neuronal differentiation in CEK cells inoculated with the same (Fig 4). The top 5 downregulated MFs in LMH cells inoculated with CSW-1 ILTV indicated catalytic activity and several binding activities, including those for structural molecules, nucleic acid and RNA; the last of which was also the only MF term downregulated in CEK cells inoculated with CSW-1 ILTV. The top 5 MF terms downregulated in LMH cells inoculated with ΔgG ILTV highlighted transporter activity, oxidoreductase activity and peptidase inhibitor activity. The top 5 CC terms in LMH cells inoculated with CSW-1 ILTV mostly included nucleus and associated components and certain protein complexes, while that inoculated with ΔgG ILTV highlighted nucleus and membrane bound organelles, and in CEK cells inoculated with CSW-1 ILTV included cytoskeletal and other structural components (Fig 4).

### 5. Pathway analysis

**Fig 5:**
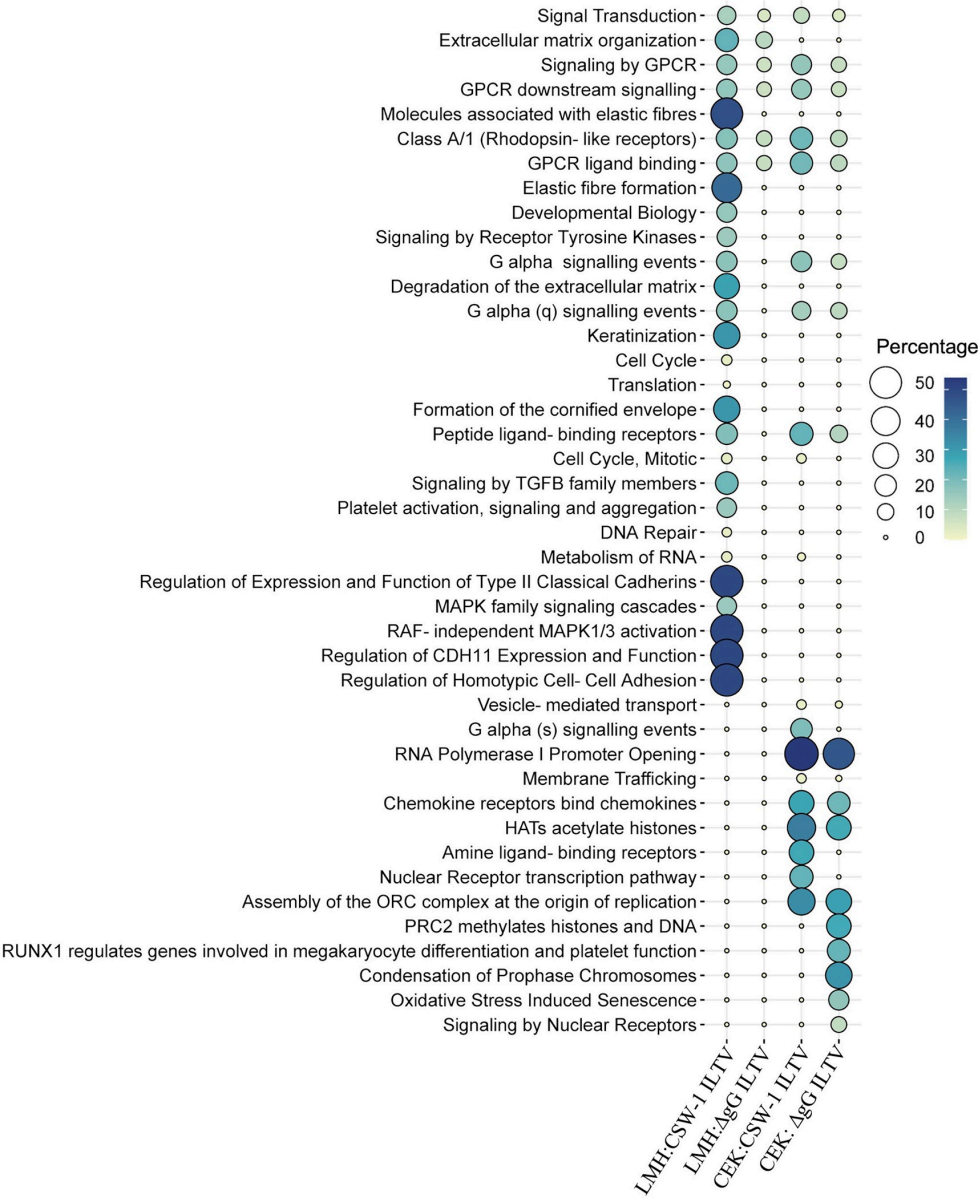
Upregulated Reactome pathways. Pathways enriched (FDR < 0.05) with the genes upregulated (Padj <0.01, log_2_ FC ≥ 1) in LMH and CEK cells inoculated with CSW-1 ILTV or ΔgG ILTV at 12 hours post inoculation are represented as bubble plots. The size and colour of bubbles are proportional to the percentage of genes enriched for the pathway.

At 12 hours post-infection with CSW-1 ILTV and ΔgG ILTV, twenty-eight and six Reactome pathways, respectively, were upregulated in LMH cells, while nineteen pathways each were upregulated in CEK cells (Fig 5). All pathways upregulated in LMH cells after ΔgG ILTV infection were also upregulated following wild-type infection, and 14 pathways in CEK cells were upregulated common to infection with both strains. Pathways related to signalling (five pathways), including ‘GPCR signalling’ and ‘Class A/1 (Rhodopsin-like receptors)’ were upregulated across all four comparisons. The pathway for ‘extracellular matrix organisation’ was upregulated uniquely in LMH cells, while those related to membrane trafficking and vesicle-mediated transport (two pathways), chemokine binding (one pathway), histone acetylation (one pathway) and transcription (two pathways) were upregulated uniquely in CEK cells, irrespective of the strain of ILTV. Pathway for ‘metabolism of RNA’ and ‘Cell Cycle, Mitotic’ were upregulated uniquely after CSW-1 ILTV infection of both cell-types.

**Fig 6:**
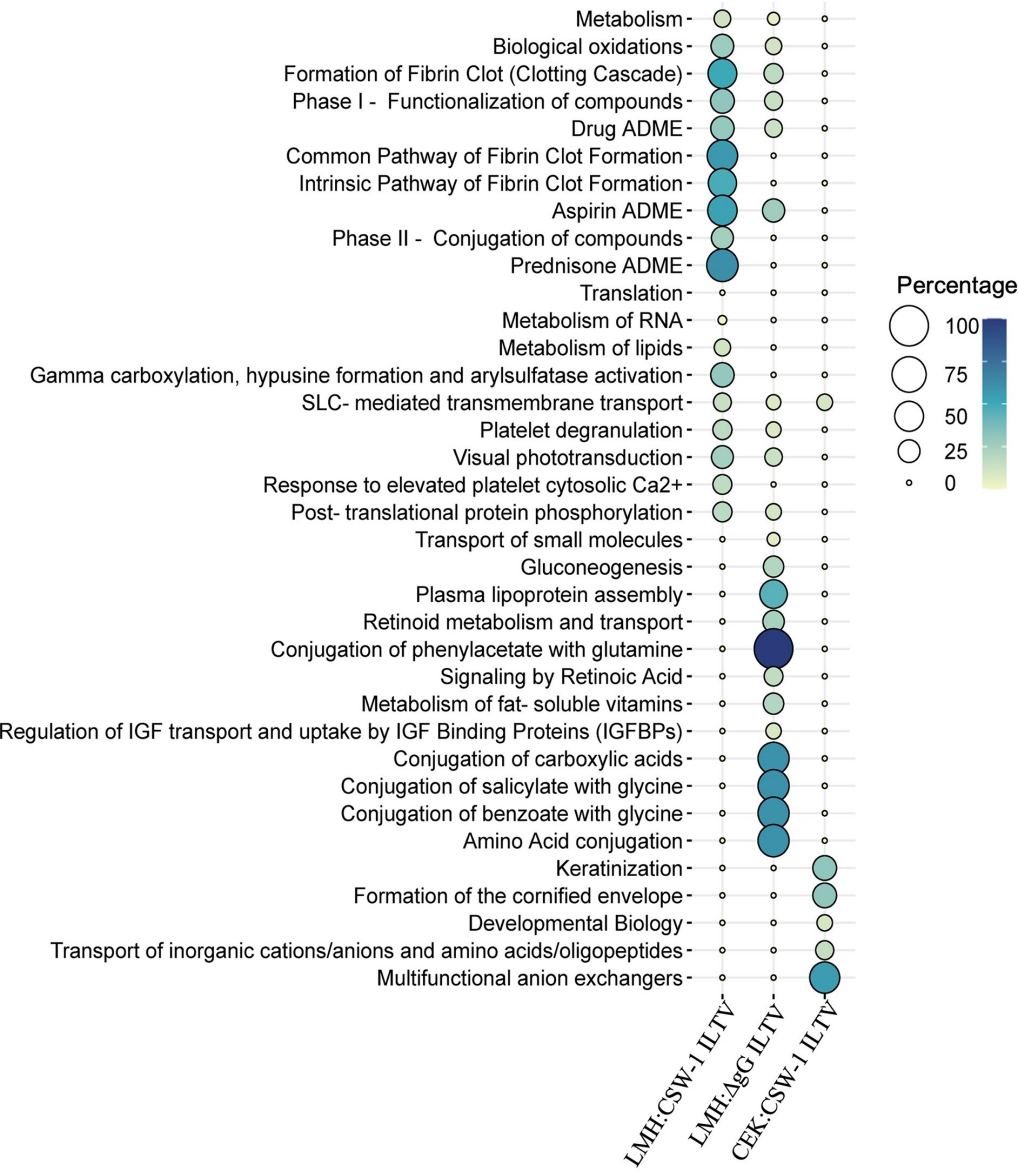
Downregulated Reactome pathways. Pathways enriched (FDR < 0.05) with the genes downregulated (Padj < 0.01, log_2_ FC ≤ -1) in LMH and CEK cells inoculated with CSW-1 ILTV or ΔgG ILTV at 12 hours post inoculation are represented as bubble plots. The size and colour of bubbles are proportional to the percentage of genes enriched for the pathway.

A total of eighteen and twenty-two Reactome pathways in LMH cells were enriched with the genes downregulated at 12 hpi with CSW-1 ILTV and ΔgG ILTV respectively (Fig 6). While six Reactome pathways were enriched with the same in CEK cells inoculated with CSW-1 ILTV, no pathways were enriched following ΔgG ILTV inoculation for the same. Among these, the pathway for ‘SLC mediated transmembrane transport’ was downregulated in all three comparisons, while those related to metabolism, biological oxidation and platelet degranulation were among the eight pathways that were downregulated uniquely in LMH cells irrespective of the strain of ILTV. Pathways involved various developmental process and transport were downregulated uniquely in CEK cells inoculated with CSW-1 ILTV.

### 6. Protein class analysis

**Fig 7:**
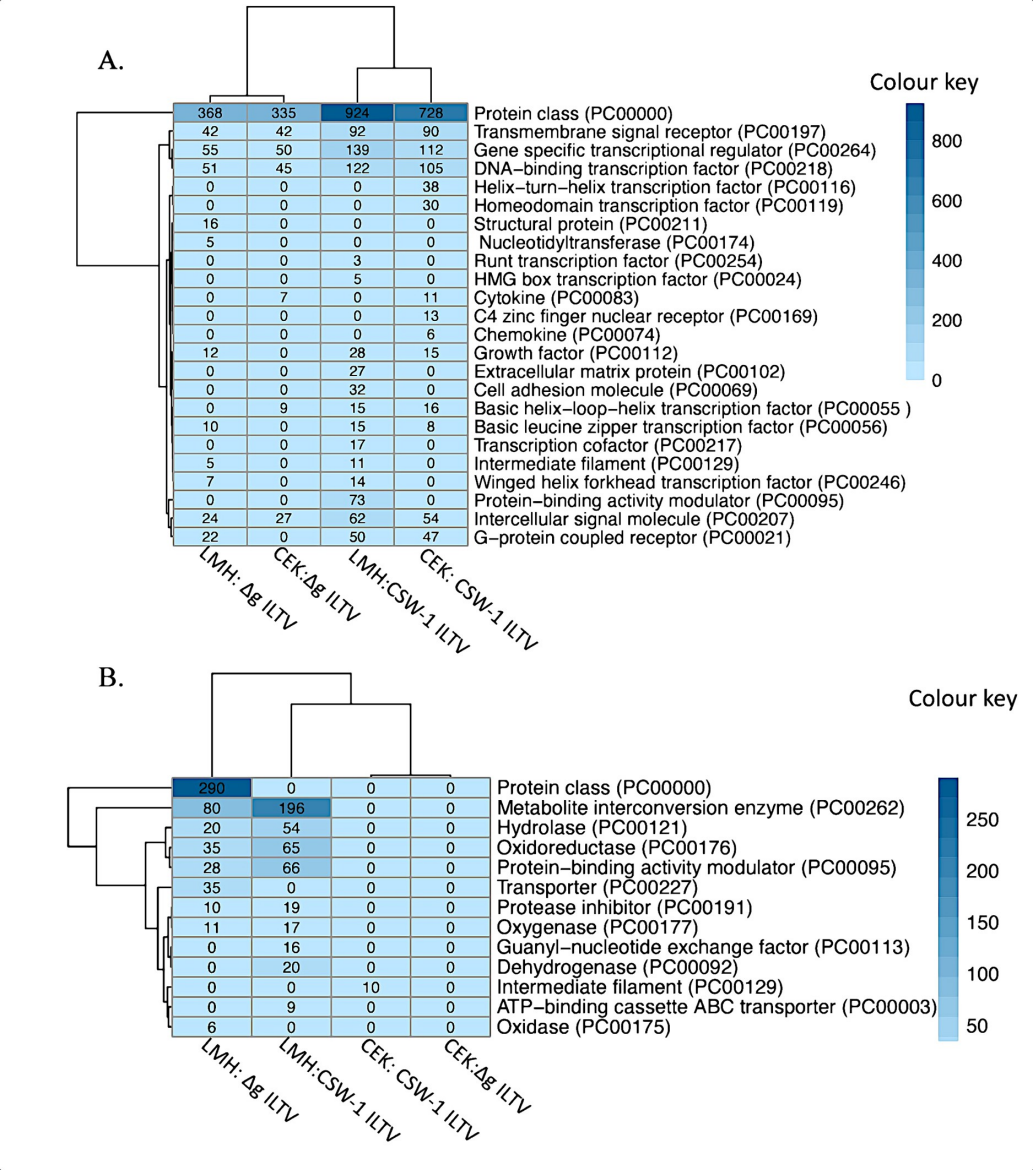
Panther protein classes. Clustered heat map representing the protein classes overrepresented (fold-change values > 1 at FDR < 0.05) with the genes that were A) upregulated or B) downregulated at 12 hours post inoculation of LMH or CEK cells, with CSW-1 ILTV or ΔgG ILTV (as compared to Mock). Numbers in the rows indicate the number of up- or down-regulated genes in the protein class. Intensity of colour reflects the number of genes in each class.

Protein class analysis of the genes that were up or downregulated after CSW-1 ILTV or ΔgG ILTV infection of LMH and CEK cells were performed using Panther 17.0. The most enriched protein classes overrepresented with fold-change values > 1 at FDR < 0.05 are represented in Fig 7. Seventeen and twelve protein classes in LMH cells and fourteen and seven protein classes in CEK cells were enriched with the genes upregulated at 12 hpi with CSW-1 ILTV and ΔgG ILTV, respectively (Fig 7A). Most of these protein classes had roles in transcriptional regulation and signalling, and were enriched across the four comparisons, indicating no cell-type specific or viral strain-specific influence in their over-representation. Interestingly, protein classes related to cytokines (PC00083) were found to be enriched only in CEK cells, irrespective of the viral strains.

Nine protein classes each were enriched with the genes downregulated after CSW-1 ILTV and ΔgG ILTV infection of LMH cells, while the same in CEK cells enriched one for the former and none for the latter, respectively (Fig 7B). Protein classes downregulated in LMH cells included those that had functional roles in various metabolic processes, transport, as well as protein binding, while the only protein class that was downregulated in CEK cells following CSW-1 ILTV inoculation was that of intermediate filament, the downregulation of which possibly indicates the damage to cytoskeletal components upon inoculation with the wild-type strain.

## Discussion

This study examined the influence of cell-type specific and virus strain-specific differences in host-virus interactions during *in vitro* ILTV infection. Transcriptome analysis of the host and ILTV were performed on the only ILTV-permissive commercial cell line (LMH cells) [28], and on CEK primary cell cultures, inoculated separately with the gG-expressing parent wild-type CSW-1 ILTV [14] and the gG-deletion mutant vaccine strain ΔgG ILTV [17,18,20].

Infection with CSW-1 ILTV (as compared to ΔgG ILTV), and infection of LMH cells (as compared to CEK cells) resulted in the differential regulation of a greater number of host genes (Table 1), while infection of CEK cells (compared to LMH cells) resulted in the differential regulation of a greater number of viral genes (Fig 2C and 2D). Apart from the gene encoding gG, the other ILTV gene differentially regulated between the two strains, commonly, in both cell types was the one encoding ORFA. The functional role of this late gene in ILTV infection is currently unclear [5,45,46]. Upregulation of this gene in the gG-expressing CSW-1 ILTV in LMH and CEK cells indicates that gG may play a role in its transcription, in both cell-types. While the expression of 12 other genes (UL [–1], UL45, US6 [gD], UL0, UL1[gL], US2, US5 [gJ], UL19, ORFC, UL54, UL31 and UL5) besides US4 (gG) and ORFA were differentially regulated in CEK cells, no significant difference was observed in the expression of any other ILTV genes in LMH cells between the two strains. This is consistent with a strong influence of cell-type in ILTV gene transcription, *in vitro*. The effect of cell-type on herpesvirus gene expression has also been reported in a previous study, where significant differences in viral gene transcription, genome amplification and genome loss were observed between different host cells inoculated *in vitro* with Epstein-Barr virus (EBV, a gammaherpesvirus) [47].

The results of ILTV gene expression in LMH cells in the current study however are in contrast with the findings from a previous study that reported differential regulation of 15 genes between these same viral strains in LMH cells at 12 hpi with 0.1 plaque-forming units (pfu) of ILTV/cell, analysed using qPCR. Of this, the expression of only five genes (ICP27, gC, gJ, Ul7 and UL40) besides gG, were differentially regulated both *in vitro* and in *vivo*, [21], reflecting differences in infection model systems. Differences in viral gene transcription between the current study and previous study are probably due to differences in transcript quantitation methods or differences in MOI used, considering the dependence of alphaherpesvirus global gene expression on viral load [48].

Of the viral genes differentially regulated in CEK cells, the majority of the up- and downregulated genes were late (7/10) and early (3/4) genes, respectively, raising the possibility for differences in replication stages between the strains in this cell type at 12 hpi. Future studies should examine viral gene expression at multiple time points after infection to investigate this further. Interestingly, of the twelve glycoproteins identified in ILTV, the expression of three (namely, gJ, gL and gD) besides gG, were upregulated in CSW-1 ILTV. Of this, gD and gL are involved in the fusion of viral envelop to host cell membrane [49–51]. Whether the close interaction of gD to gL/gH complex on viral envelope has a role in their co-upregulation, however, is not clear. The upregulated genes gJ and UL0, and the downregulated gene ORFC are associated with ILTV virulence [52,53]. Previous studies of the deletion mutant strains of ILTV, deficient in UL0, gJ or ORFC separately, have demonstrated marked attenuation in *vivo*, indicating their suitability as vaccine candidates [53–56]. Although the upregulation of the gene for UL0 and gJ in the CSW-1 ILTV inoculated CEK cells in the current study aligns with the virulence phenotype of the wild-type strains [18], Mahmoudian *et al*. observed a greater transcription of the latter in ΔgG ILTV inoculated LMH cells and *in vivo*, compared to the wild-type strain [21]. Differential regulation of the UL[–1] gene was similar between the current study and the study conducted by Mahmoudian *et al*. (2013), where a greater expression was observed for the gene in CEK cells in the current study, and *in vivo* (but not in LMH cells) in the previous study, following CSW-1 ILTV inoculation [21]. Given the predicted role of UL[–1] in cleavage and encapsidation of ILTV DNA, modulation of host cell gene expression, and as structural constituents of viral nucleocapsids [57], upregulation of this gene in the wild-type ILTV may contribute to its virulence, as suggested by Mahmoudian *et al*. [21].

Of the several pathways upregulated with the host genes, the pathway for ‘HATs acetylate histone’ in CEK cells inoculated with either strain of ILTV (Fig 5), and the gene encoding DOT1L (log_2_FC = 1.01847607) involved in histone methylation, upregulated in LMH cells inoculated with CSW-1 ILTV, indicated histone modifications, consistent with herpes simplex virus 1 (HSV-1) lytic replication [58–60]. Inhibition of histone methylation via DOT1L blockage or by other means, during HSV-1, human cytomegalovirus (HCV) or avian leucosis virus-J infection, restricted viral replication in several *in vitro* studies [58,61,62]. In some cases, DOT1L inhibition also restored virally-suppressed interferon (IFN) and interferon stimulated gene (ISG) functions [62] or alleviated pathology [63], indicating the role of this gene in facilitating viral replication, immune augmentation and promoting pathology following viral infection. Furthermore, dynamic changes in histone methylations specific to Marek’s disease virus (MDV) resistant and susceptible chickens during MDV infection have been demonstrated, emphasizing the role of intrinsic epigenetic mechanisms in the control of the infection [64]. Despite the direct correlation between histone acetylation and the expression of ICP0 [59], no difference was observed in the expression of this gene in either cell-type, but the gene encoding the histone deacetylase, HDAC11 (log_2_FC = -1.5120437) [65] was downregulated in CEK cells inoculated with CSW-1 ILTV. Modulation of histone deacetylation via the inhibition of HDAC1 or HDAC2 activity by alphaherpesviruses including MDV, surpass the repression of viral gene expression and/or interfere with host immune responses to facilitate efficient viral replication [66–70]. While not much is known about the functions of this recently discovered histone deacetylase (HDAC11), a recent study demonstrated significant reduction in the expression of interferon-stimulated genes in the absence of HDAC11 expression during influenza virus (IAV) infection, attributing anti-viral functions to this enzyme [65]. Given the relevance of DOT1L upregulation and HDAC11 inhibition during viral infections, the up- and down-regulation of these genes respectively, during ILTV infection in this study, suggests their likely involvement in ILTV mediated immune augmentation, viral replication or pathology, that demands detailed further investigations.

Downregulation of several endosomal TLRs (TLR3, 7 and 21 after CSW-1 ILTV infection and TLR7 after ΔgG ILTV) in LMH cells, was consistent with the results of a previous study that reported a reduction in mRNA levels for TLR3 and TLR7, and an inconclusive level of expression of TLR21 in LMH cells inoculated with ILTV [71]. Differential regulation of TLRs between LMH and CEK cells in the current study (Table 3), could have contributed to the differences in cytokine/chemokine gene transcription between them, considering the relevance of TLR induction in the regulation of cytokine and chemokine gene expression and the subsequent initiation of immune responses [72]. Upregulation of a greater number of C-C and C-X-C family of cytokine/chemokine encoding genes in CEK cells as compared to LMH cells (Table 2), also suggested a distinct pattern of expression of these genes in the two cell types, which agrees with several previous studies that identified unique cytokine/chemokine signatures in different cell types during *in vitro* stimulations or infection with the same pathogen [73–76]. The expression levels of cytokine and chemokine genes were relatively higher during infection with the gG-expressing CSW-1 ILTV compared to ΔgG ILTV, in both cell types (Table 2). This included the expression of the genes encoding CXCL14 and IL-8 orthologues (chCXCLi1 and chCXCLi2), which contrasted with findings from Coppo *et al*., that observed higher expression of these transcripts in the absence of ILTV gG in TOCs and or blood derived monocytes (BDMs) at different timepoints, analysed using qPCR [22]. These differences between studies likely reflect the different infection systems used, as well as the differences in timepoints across studies, and possibly the different methods of transcript quantification.

Consistent with the upregulation of chemokine and cytokine encoding genes, GO terms indicating chemotaxis were upregulated in all four comparisons. Interestingly, the GO term for ‘cell migration’ was also downregulated in CEK cells inoculated with CSW-1 ILTV (FDR 1.75E-02), and comprised 27 genes including those encoding several members of the semaphorin family of immunoregulators such as Semaphorin 4D, Semaphorin 4G and Semaphorin 7A, laminins such as LAMa3,and LAMb3, Ephrin-A1 and TIAM-1, with roles in the maintenance of immunological homeostasis, CD4+ T cell mediated chemotaxis and T cell transendothelial migration [77–80], in humans. Downregulation of these genes and the corresponding GO term, in the gG expressing CSW-1 ILTV infected cells, is consistent with the immunomodulatory role of gG [17,18,20,22]. Whether these genes play a role in immune response to ILTV infection in chickens, and whether gG influences their expression or functions *in vivo*, is currently unknown and warrants additional research. One of the other sets of genes with immunoregulatory functions, upregulated in this study were the SOCS family members (SOCS 1, 2 and 3 in LMH cells inoculated with either strain, SOCS1 and 2 in CSW-1 ILTV- inoculated CEK cells and SOCS1 in ΔgG ILTV-inoculated CEK cells). Upregulation of these genes has been observed during infection with herpesviruses such as HSV-1, MDV and ILTV, where their increased expression during infection with the former two have been correlated to immunosuppression or susceptibility to infection [27,81,82].

A previous study reported upregulation of metabolic processes at 12 hpi with a virulent ILTV strain (LJS09) in LMH cells and primary cell cultures. Although not in the top 5 list of enriched gene ontologies, metabolic processes for organic substances, including nucleobases were enriched with both the up and downregulated genes, in both cell-types in the current study after ILTV infection. These differences between the studies could be due to differences in viral strains and the MOI used for inoculation as suggested previously for the different strains of Newcastle Disease Virus (NDV) [83].

In agreement with the critical role of the protooncogene FOS in ILTV genome replication and virion production [30], genes encoding several FOS family members (FOSB, FOS and/or FOSL2), were upregulated in both cell-types infected with either strain. Genes upregulated for the BCL-2 family of antiapoptotic proteins (MCL1 in LMH cells, and BCL2A and BCL2L14 in CEK cells infected with either strain of ILTV) [84,85], were in agreement with the notion that ILTV infection prevents apoptosis of infected cells, in order to prolong viral replication [11]. Being a carcinoma cell line, LMH cells also seemed to possess intrinsic mechanisms for cell cycle regulation, that was evidenced by the upregulation of the pathway for TGF-β signalling and the downregulation of the gene encoding TRAIL-like, uniquely in the tumour cell line (not in CEK cells). TGF-β signalling and TRAIL-like have distinct, but opposite roles in normal and tumour cells [86–88]. TGF-β signalling induces apoptosis of normal cells, but selectively promotes the growth of tumour cells such as LMH cells [87,88], while TRAIL-like brings about the opposite effect in these two cell-types [86]. Hence, the anti-apoptotic effect generated in LMH cells during ILTV infection appeared to be multifactorial, determined by both tumour-cell specific effects, and the immunomodulatory effects of ILTV in preventing infected cell apoptosis to ensure persistent replication [11].

Downregulation of several TLRs in LMH cells agrees with the results of a previous study that indicated the non-suitability of this cell line for the study of TLR responses during ILTV infection [71]. Furthermore, it has also been speculated that several cellular internal pathways related to NF-kB signalling and other proteins are dysregulated in this cell line [71]. Given these findings, it is compelling to hypothesize that the differential regulation of TLRs between the cell-types, and the inherent dysregulation of signalling pathways in the carcinoma cell line, LMH, contributed greatly to some of the major differences in host and viral gene transcription between the two cell types in the current study. Our knowledge on the association of ILTV to histones is limited to latency establishment, and the association of ILTV VP22 to histones that influences cell cycle regulation [89]. Recent research underscores the necessity to investigate the impact of histone modifications during the lytic phase of ILTV replication, as this may have a role in viral replication or ILTV-induced immune modulations,similar to what has been reported for other herpesviruses [60,63,64,66–70]. Several genes with immunomodulatory functions, differentially regulated between cells infected with the gG expressing wild-type and gG-deletion mutant ΔgG ILTV was consistent with the role of gG in immune augmentation. Nevertheless, the role of these genes during ILTV infection requires verification *in vivo*.

## Conclusion

Taken together, results from this study indicate that the differential regulation of host and ILTV genes during *in vitro* infection were largely governed by the differences in cell-types, and to a lesser extent, by the presence or absence of gG. These results highlight the importance of selecting appropriate cell culture systems for *in vitro* experiments, and the importance of characterising viruses in multiple different cell types where possible. A future goal will be to study the dynamics of host-virus interactions *in vivo*, focusing on the identification and characterization of specific genes or pathways associated with ΔgG ILTV attenuation and vaccine efficacy.

## Supporting information

S1 Fig Global gene expression pattern of host genes. A) Principal component analysis (PCA) plot and B) Sample-to-sample distances plot of DESeq2 differential gene expression analysis of chicken genes. M1-3_C, ΔgG1-3_C and C1-3_C denotes biological replicates of the Mock, ΔgG ILTV and CSW-1 ILTV groups in CEK cells respectively. M1-3_L, ΔgG1-3_L and C1-3_L denotes biological replicates of the Mock, ΔgG ILTV and CSW-1 ILTV group in LMH cells respectively. Relationships between samples (B) are indicated by clustering, reflected by the intensity of colour in the squares shared by samples; darker colour indicates more correlation, and lighter colour indicates less correlation.
(TIF)

S2 Fig Global gene expression pattern of ILTV genes. A) Principal component analysis (PCA) plot and B) Sample-to-sample distances plot of the DESeq2 differential gene expression analysis of ILTV genes. ΔgG1-3_C and C1-3_C denotes biological replicates of the ΔgG ILTV and CSW-1 ILTV inoculated groups in CEK cells, respectively while ΔgG1-3_L and C1-3_L denotes biological replicates of the ΔgG ILTV and CSW-1 ILTV inoculated groups in LMH cells respectively. Relationships between samples (B) are indicated by clustering, reflected by the intensity of colour in the squares shared by samples; darker colour indicates more correlation, and lighter colour indicates less correlation.
(TIF)

S1 Table Statistical comparison of the genome copy number per reaction of ILTV UL15 DNA in the supernatant samples collected after mock inoculation or inoculation with CSW-1 ILTV or ΔgG ILTV in LMH and CEK cells at an MOI of 3.5. (DOCX)

S2 Table Summary of the reads of LMH or CEK cells 12 hours after mock-inoculation or inoculation with CSW-1 ILTV or ΔgG ILTV, mapped to the chicken or ILTV genome. (DOCX)

S3 Table Top 10 upregulated host genes in LMH cells at 12 hours post-inoculation with CSW-1 or ΔgG ILTV. (DOCX)

S4 Table Top 10 upregulated host genes in CEK cells at 12 hours post-inoculation with CSW-1 or ΔgG ILTV. (DOCX)

S5 Table Top 10 downregulated host genes in LMH cells at 12 hours post-inoculation with CSW-1 or ΔgG ILTV. (DOCX)

S6 Table Top 10 downregulated host genes in CEK cells at 12 hours post-inoculation with CSW-1 or ΔgG ILTV. (DOCX)

S7 Table Gene ontologies enriched with the genes upregulated in LMH cells at 12 hours post inoculation with CSW-1 ILTV. (XLSX)

S8 Table Gene ontologies enriched with the genes upregulated in LMH cells at 12 hours post inoculation with ΔgG ILTV. (XLSX)

S9 Table Gene ontologies enriched with the genes upregulated in CEK cells at 12 hours post inoculation with CSW-1 ILTV. (XLSX)

S10 Table Gene ontologies enriched with the genes upregulated in CEK cells at 12 hours post inoculation with ΔgG ILTV. (XLSX)

S11 Table Gene ontologies enriched with the genes downregulated in LMH cells at 12 hours post inoculation with CSW-1 ILTV. (XLSX)

S12 Table Gene ontologies enriched with the genes downregulated in LMH cells at 12 hours post inoculation with ΔgG ILTV. (XLSX)

S13 Table Gene ontologies enriched with the genes downregulated in CEK cells at 12 hours post inoculation with CSW-1 ILTV. (XLSX)

S14 Table Upregulated host gens in LMH cells at 12 hours post inoculation with CSW-1 ILTV. (XLSX)

S15 Table Upregulated host gens in LMH cells at 12 hours post inoculation with ΔgG ILTV. (XLSX)

S16 Table Upregulated host gens in CEK cells at 12 hours post inoculation with CSW-1 ILTV. (XLSX)

S17 Table Upregulated host gens in CEK cells at 12 hours post inoculation with ΔgG ILTV. (XLSX)

S18 Table Downregulated host gens in LMH cells at 12 hours post inoculation with CSW-1 ILTV. (XLSX)

S19 Table Downregulated host gens in LMH cells at 12 hours post inoculation with ΔgG ILTV. (XLSX)

S20 Table Downregulated host gens in CEK cells at 12 hours post inoculation with CSW-1 ILTV. (XLSX)

S21 Table Downregulated host gens in CEK cells at 12 hours post inoculation with ΔgG ILTV. (XLSX)
