# Supplementary figures and images for: Transcriptomic analyses of host-virus interactions during in vitro infection with wild-type and glycoprotein g-deficient (ΔgG) strains of ILTV in primary and continuous cell cultures
Source: PLoS One. 2024 Oct 11;19(10):e0311874. doi: 10.1371/journal.pone.0311874 (PMC11469545; doi:10.1371/journal.pone.0311874)

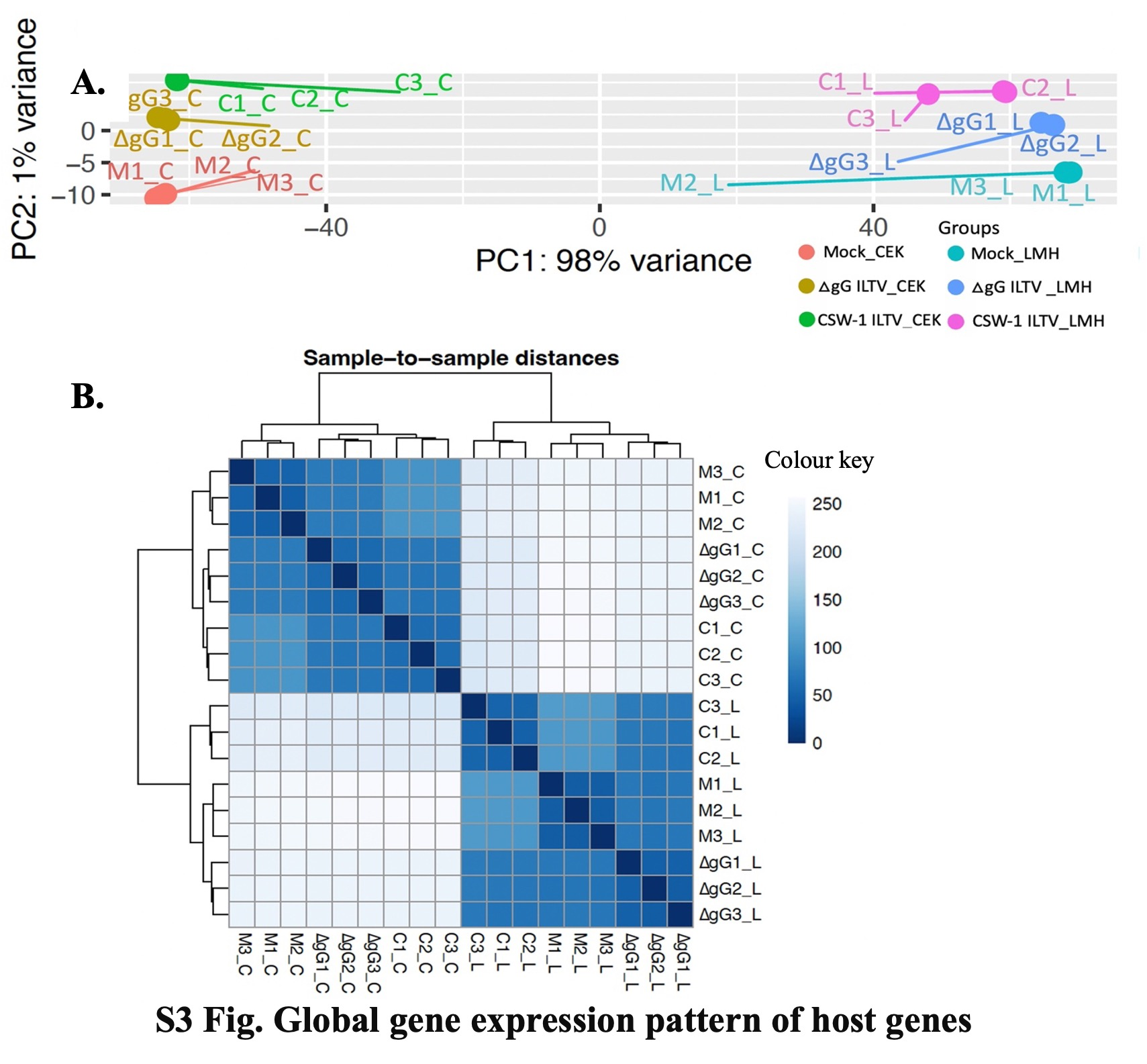

Supplement: S1 Fig — A) Principal component analysis (PCA) plot and B) Sample-to-sample distances plot of DESeq2 differential gene expression analysis of chicken genes. M1-3_C, ΔgG1-3_C and C1-3_C denotes biological replicates of the Mock, ΔgG ILTV and CSW-1 ILTV groups in CEK cells respectively. M1-3_L, ΔgG1-3_L and C1-3_L denotes biological replicates of the Mock, ΔgG ILTV and CSW-1 ILTV group in LMH cells respectively. Relationships between samples (B) are indicated by clustering, reflected by the intensity of colour in the squares shared by samples; darker colour indicates more correlation, and lighter colour indicates less correlation. (TIF) [file pone.0311874.s001.tif]

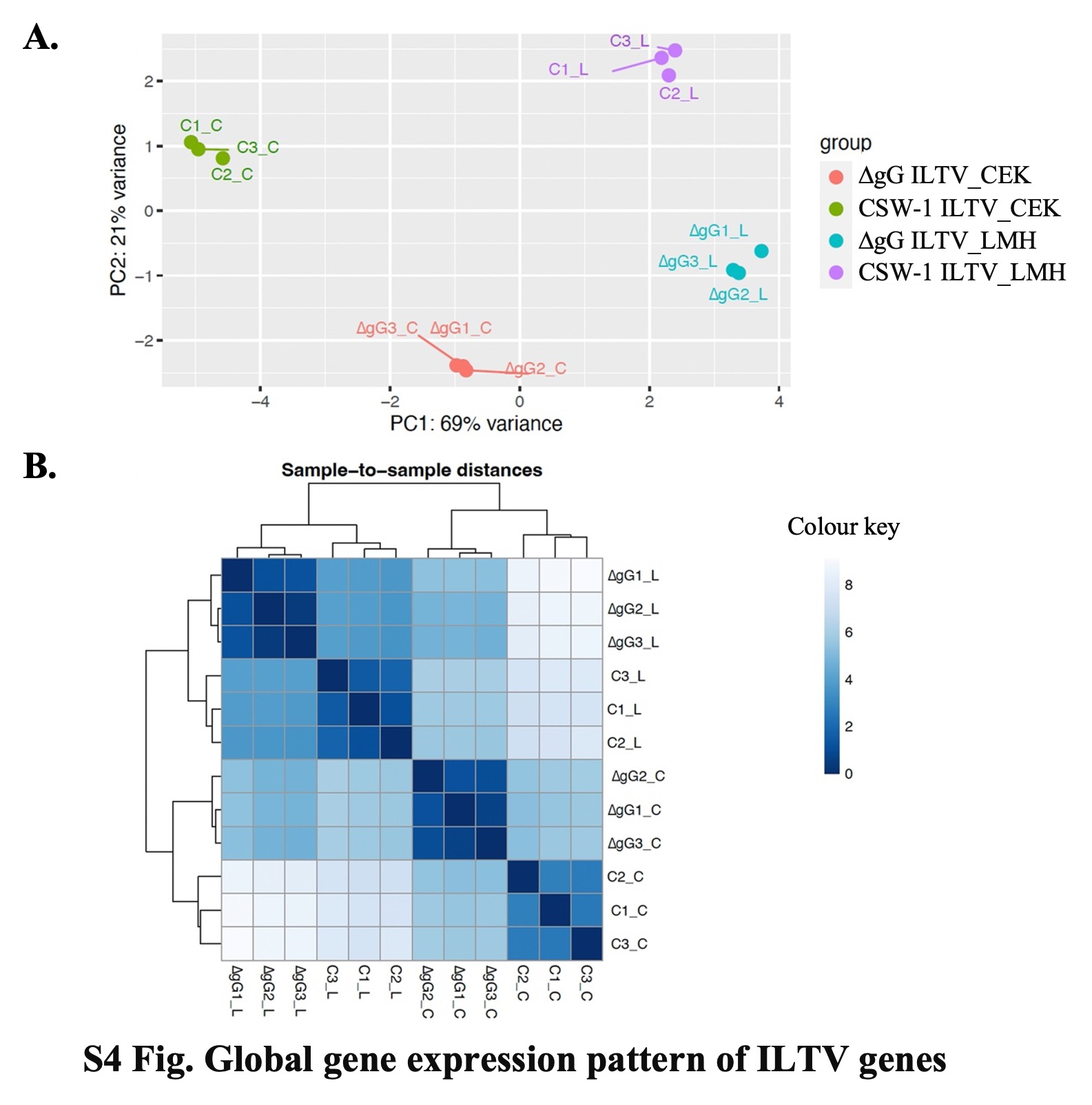

Supplement: S2 Fig — A) Principal component analysis (PCA) plot and B) Sample-to-sample distances plot of the DESeq2 differential gene expression analysis of ILTV genes. ΔgG1-3_C and C1-3_C denotes biological replicates of the ΔgG ILTV and CSW-1 ILTV inoculated groups in CEK cells, respectively while ΔgG1-3_L and C1-3_L denotes biological replicates of the ΔgG ILTV and CSW-1 ILTV inoculated groups in LMH cells respectively. Relationships between samples (B) are indicated by clustering, reflected by the intensity of colour in the squares shared by samples; darker colour indicates more correlation, and lighter colour indicates less correlation. (TIF) [file pone.0311874.s002.tif]
