# Supplementary material for: Transcriptomic analyses of host-virus interactions during in vitro infection with wild-type and glycoprotein g-deficient (ΔgG) strains of ILTV in primary and continuous cell cultures
Source: PLoS One. 2024 Oct 11;19(10):e0311874. doi: 10.1371/journal.pone.0311874 (PMC11469545; doi:10.1371/journal.pone.0311874)
Supplement: S1 Table — (DOCX) [file pone.0311874.s003.docx]

**Table S1. Statistical comparison of the genome copy number per reaction of ILTV UL15 DNA in the supernatant samples**

**collected after mock inoculation or inoculation with CSW-1 ILTV or ∆gG ILTV in LMH and CEK cells at an MOI of 3.5.**

| Time post inoculation | | *p-value |
| --- | --- | --- |
| 0hpi | 0hpi |  |
| LMH: CSW-1ILTV | LMH: ∆gG ILTV | 0.1568 |
| LMH: CSW-1ILTV | CEK: CSW-1 ILTV | 0.2547 |
| LMH: ∆gG ILTV | CEK: ∆gG ILTV | 0.1206 |
| CEK: CSW-1 ILTV | CEK: ∆gG ILTV | 0.6730 |
| 12hpi | 12hpi |  |
| LMH: CSW-1ILTV | LMH: ∆gG ILTV | 0.2176 |
| LMH: CSW-1ILTV | CEK: CSW-1 ILTV | 0.0417 |
| LMH: ∆gG ILTV | CEK: ∆gG ILTV | 0.0250 |
| CEK: CSW-1 ILTV | CEK: ∆gG ILTV | 0.0858 |
| 0hpi | 12hpi |  |
| LMH: CSW-1ILTV | LMH: CSW-1ILTV | < 0.0001 |
| LMH: ∆gG ILTV | LMH: ∆gG ILTV | 0.014 |
| CEK: CSW-1 ILTV | CEK: CSW-1 ILTV | 0.0023 |
| CEK: ∆gG ILTV | CEK: ∆gG ILTV | < 0.0001 |

*p-value < 0.05 was considered significantly different.
