# Supplementary material for: Transcriptomic analyses of host-virus interactions during in vitro infection with wild-type and glycoprotein g-deficient (ΔgG) strains of ILTV in primary and continuous cell cultures
Source: PLoS One. 2024 Oct 11;19(10):e0311874. doi: 10.1371/journal.pone.0311874 (PMC11469545; doi:10.1371/journal.pone.0311874)
Supplement: S2 Table — (DOCX) [file pone.0311874.s004.docx]

**Table S2.** **Summary of the reads of LMH or CEK cells 12 hours after mock-inoculation or inoculation with CSW-1 ILTV or ∆gG ILTV, mapped to the chicken or ILTV genome.**

| Cell type | Group | Total mapped reads (%) | Reads mapped to chicken genome (%) | Reads mapped to ILTV genome (%) |
| --- | --- | --- | --- | --- |
| LMH | CSW-1 ILTV-1 ^*^ | 4.15E+07 (59.8) | 2.95E+07 (42.0) | 1.20E+07 (17.8) |
|  | CSW-1 ILTV-2 ^*^ | 3.73E+07 (59.1) | 2.74E+07 (42.6) | 9.90E+06 (16.5) |
|  | CSW-1 ILTV-3 ^*^ | 3.39E+07 (58.3) | 2.41E+07 (40.8) | 9.80E+06 (17.5) |
|  | ∆gG^-^ ILTV-1 ^†^ | 3.19E+07 (64.5) | 3.02E+07 (60.8) | 1.70E+06 (3.7) |
|  | ∆gG- ILTV-2 ^†^ | 4.22E+07 (63.8) | 4.01E+07 (62.6) | 2.10E+06 (3.6) |
|  | ∆gG- ILTV-3 ^†^ | 4.64E+07 (66.8) | 4.39E+07 (62.9) | 2.50E+06 (3.9) |
|  | MOCK-1^¶^ | 4.93E+07 (67.5) | 4.93E+07 (67.5) | nil |
|  | MOCK-2^¶^ | 3.50E+07 (60.6) | 3.50E+07 (60.6) | nil |
|  | MOCK-3^¶^ | 3.83E+07 (61.0) | 3.83E+07 (61.0) | nil |
| CEK | CSW-1 ILTV-1 ^*^ | 4.17E+07 (56.0) | 2.06E+07 (28.3) | 2.11E+07 (27.7) |
|  | CSW-1 ILTV-2 ^*^ | 2.69E+07 (57.2) | 1.37E+07 (30.6) | 1.32E+07 (26.6) |
|  | CSW-1 ILTV-3 ^*^ | 4.13E+07 (58.9) | 2.04E+07 (30.5) | 2.09E+07 (28.4) |
|  | ∆gG^-^ ILTV-1 ^†^ | 3.17E+07 (63.0) | 2.58E+07 (50.8) | 5.90E+06 (12.2) |
|  | ∆gG- ILTV-2 ^†^ | 3.67E+07 (62.9) | 3.03E+07 (51.4) | 6.40E+06 (11.5) |
|  | ∆gG- ILTV-3 ^†^ | 3.64E+07 (66.6) | 3.02E+07 (54.7) | 6.20E+06 (11.3) |
|  | MOCK-1^¶^ | 3.75E+07 (66.4) | 3.75E+07 (66.4) | nil |
|  | MOCK-2^¶^ | 4.95E+07 (66.6) | 4.95E+07 (66.6) | nil |
|  | MOCK-3^¶^ | 1.96E+07 (61.5) | 1.96E+07 (61.5) | nil |

Biological replicates of the ^*^CSW-1 ILTV inoculated group; ^†^ ∆gG ILTV inoculated group and the ^¶^ the Mock inoculated group
