# Supplementary material for: Transcriptomic analyses of host-virus interactions during in vitro infection with wild-type and glycoprotein g-deficient (ΔgG) strains of ILTV in primary and continuous cell cultures
Source: PLoS One. 2024 Oct 11;19(10):e0311874. doi: 10.1371/journal.pone.0311874 (PMC11469545; doi:10.1371/journal.pone.0311874)
Supplement: S3 Table — (DOCX) [file pone.0311874.s005.docx]

**Table S5. Top 10 upregulated host genes in LMH cells at 12 hours post-inoculation with CSW-1 or ∆gG ILTV.**

| **CSW-1 ILTV vs Mock** |  | **∆gG ILTV vs Mock** | |  |
| --- | --- | --- | --- | --- |
| **Gene name** | **log_2_FC*** | **Gene name** | **log_2_FC** | |
| ***Osteoblast survival, cell fate determination*** | | | | |
| Mab-21 like 1 | 10.33 | Mab-21 like 1 | 8.31 | |
| ***Cell adhesion*** | | | | |
| Protocadherin 8 | 9.8 | Protocadherin 8 | 6.84 | |
| ***Regulation of transcription*** | | | | |
| Early growth response 4 | 8.57 | Early growth response 4 | 6.25 | |
| Early growth response 3 | 7.61 |  |  | |
| ***Extracellular remodelling*** | | | | |
| Activity regulated cytoskeleton associated protein | 8.84 | Activity regulated cytoskeleton associated protein | 5.62 | |
| ADAM metallopeptidase with thrombospondin type 1 motif | 7.88 |  |  | |
| ***Signalling*** | | | | |
| Melanocortin 2 receptor | 8.48 | Melanocortin 2 receptor | 6.05 | |
| ***Metabolism*** | | | | |
| Prostaglandin-endoperoxide synthase 2 | 8.03 | Prostaglandin-endoperoxide synthase 2 | 5.1 | |
| ***Immune response*** | | | | |
| Immunoglobulin superfamily member 1 | 7.46 | Immunoglobulin superfamily member 1 | 5.44 | |
|  |  | Interleukin 8 | 5.10 | |
| ***Transport*** | | | | |
|  |  | Potassium channel, subfamily K, member 4 | 5.28 | |
|  |  | Fatty acid binding protein 1 | 5.08 | |
| ***Structural constituent of neuronal cytoskeleton*** | | | | |
| Neurofilament heavy polypeptide (NEFH) | 8.07 |  | | |
|  |  |  |  | |

Padj < 0.01, log_2_FC ≥ 1 was considered significant; *log_2_FC, log_2_ fold change
