# Supplementary material for: Transcriptomic analyses of host-virus interactions during in vitro infection with wild-type and glycoprotein g-deficient (ΔgG) strains of ILTV in primary and continuous cell cultures
Source: PLoS One. 2024 Oct 11;19(10):e0311874. doi: 10.1371/journal.pone.0311874 (PMC11469545; doi:10.1371/journal.pone.0311874)
Supplement: S4 Table — (DOCX) [file pone.0311874.s006.docx]

**Table S6. Top 10 upregulated host genes in CEK cells at 12 hours post-inoculation with CSW-1 or ∆gG ILTV.**

| **CSW-1 ILTV vs Mock**  **Gene name** | | | **log_2_FC*** | | | **∆gG ILTV vs Mock**  **Gene name** | | | **log_2_FC** |
| --- | --- | --- | --- | --- | --- | --- | --- | --- | --- |
| ***Regulation of transcription/ translation*** | | | | | | | | | |
| Nuclear receptor subfamily 2 group E member 1 | 9.03 | | | Nuclear receptor subfamily 2 group E member 1 | | | 7.84 | | |
| Basic helix-loop-helix family member e23 | 7.26 | | | Basic helix-loop-helix family member e23 | | | 5.85 | | |
| Zinc finger protein 488 | 6.82 | | |  | | |  | | |
| Nuclear receptor subfamily 4 group A member 2 | 6.05 | | |  | | |  | | |
| ***Signalling/signal transduction*** | | | | | | | | | |
| Ras related dexamethasone induced 1 | 7.80 | | | Ras related dexamethasone induced 1 | | | 6.71 | | |
| Adrenoceptor beta 2 | 7.41 | | | Zinc finger protein 488 | | | 6.48 | | |
| Arginine vasopressin receptor 1A | 6.45 | | | Adrenoceptor beta 2 | | | 6.07 | | |
| ***Structural constituent of cytoskeleton*** | | | | | | | | | |
| Feather keratin 1-like | 5.96 | | | Feather keratin 1-like | | | 7.68 | | |
|  |  | | | Feather keratin 3-like | | | 6.47 | | |
|  |  | | | *Gallus gallus* feather keratin 3-like (LOC100859191) | | | 6.07 | | |
| ***Cell adhesion*** | | | | | | | | | |
| Protocadherin 8 | 8.95 | | | Protocadherin 8 | | | 7.37 | | |
| ***Immune response*** | | | | | | | | | |
|  |  | | | Lymphoid-restricted membrane protein-like | | | 5.21 | | |
| ***Heme binding/catabolic process*** | | | | | | | | | |
| Eye-globin | | 6.44 | | |  | | | | |
|  |  |  | | |  | | |  | |

Padj < 0.01, log_2_FC ≥ 1 was considered significant; *log_2_FC, log_2_ fold change
