# Supplementary material for: Transcriptomic analyses of host-virus interactions during in vitro infection with wild-type and glycoprotein g-deficient (ΔgG) strains of ILTV in primary and continuous cell cultures
Source: PLoS One. 2024 Oct 11;19(10):e0311874. doi: 10.1371/journal.pone.0311874 (PMC11469545; doi:10.1371/journal.pone.0311874)
Supplement: S5 Table — (DOCX) [file pone.0311874.s007.docx]

**Table S7. Top 10 downregulated host genes in LMH cells at 12 hours post-inoculation with CSW-1 or ∆gG ILTV.**

| **CSW-1 ILTV vs Mock** |  | **∆gG ILTV vs Mock** |  | |
| --- | --- | --- | --- | --- |
| **Gene name** | **log_2_FC*** | **Gene name** | | **log_2_FC** |
| ***Metabolism*** | | | | |
| Cytochrome P450 family 7 subfamily A member 1 | -6.90 | Cytochrome P450 family 7 subfamily A member 1 | | -4.84 |
| Angiopoietin like 3 | -5.53 | Acyl-CoA synthetase medium-chain family member 4 | | -3.46 |
| Glucose-6-phosphatase catalytic subunit | -5.01 | Phosphoenolpyruvate carboxykinase 1 | | -3.42 |
| Ethanolamine-phosphate phospho-lyase | -4.58 | Cytochrome P450, family 4, subfamily B, polypeptide 7 | | -3.33 |
|  |  | Angiopoietin like 3 | | -3.19 |
|  |  | Xanthine dehydrogenase | | -3.12 |
|  |  | Glucose-6-phosphatase catalytic subunit | | -3.12 |
|  |  | Fatty acid binding protein 2 | | -2.91 |
| ***Signalling/signal transduction*** | | | | |
| Glucosaminyl (N-acetyl) transferase 2, I-branching enzyme | -5.63 | Cholinergic receptor nicotinic beta 4 subunit | | -3.62 |
| Opioid related nociceptin receptor 1 | -4.69 | Glucosaminyl (N-acetyl) transferase 2, I-branching enzyme (I Blood group) | | -3.5 |
| G protein-coupled receptor 183 | -4.47 |  | |  |
| ***Regulation of transcription*** | | | | |
| Neuronal differentiation 6 | -6.10 |  | |  |
| ***Transport*** | | | | |
| PDZ domain containing 1 pseudogene 1 | -4.79 |  | |  |
| ***Cell adhesion*** | | | | |
| Claudin 2 | -4.56 |  | | |
|  |  |  | |  |

Padj < 0.01, log_2_FC ≤ -1 was considered significant; *log_2_FC, log_2_ fold change
