# Supplementary material for: Transcriptomic analyses of host-virus interactions during in vitro infection with wild-type and glycoprotein g-deficient (ΔgG) strains of ILTV in primary and continuous cell cultures
Source: PLoS One. 2024 Oct 11;19(10):e0311874. doi: 10.1371/journal.pone.0311874 (PMC11469545; doi:10.1371/journal.pone.0311874)
Supplement: S6 Table — (DOCX) [file pone.0311874.s008.docx]

**Table S8. Top 10 downregulated host genes in CEK cells at 12 hours post-inoculation with CSW-1 or ∆gG ILTV.**

| **CSW-1 ILTV vs Mock** |  | **∆gG ILTV vs Mock** |  | |
| --- | --- | --- | --- | --- |
| **Gene name (Gene symbol)** | **log_2_FC*** | **Gene name (Gene symbol)** | | **log_2_fc** |
| ***Regulation of transcription/ translation*** | | | | |
| LIM homeobox transcription factor 1, alpha | -3.45 | Chromosome 4 open reading frame, human | | -1.46 |
| Tripartite motif containing 2 | -3.18 | RBBP8 N-terminal like | | -1.39 |
| RBBP8 N-terminal like | -3.05 |  | |  |
| Zinc finger protein 750 | -2.83 |  | |  |
| ***Signalling/signal transduction*** | | | | |
| SLIT and NTRK like family member 6 | -3.68 | SLIT and NTRK like family member 4 | | -1.45 |
|  |  |  | |  |
| ***Transport*** | | | | |
| Solute carrier family 26 member 3 | -3.20 | Solute carrier family 26 member 9 | | -1.34 |
| Small integral membrane protein 5 | -2.94 |  | |  |
| ***Structural component*** | | | | |
| Keratin-associated protein 9-1-like | -3.47 |  | |  |
| ***Stress response*** | | | | |
| Heat shock protein family B (small) member 2 | -2.87 |  | |  |
| ***Cell proliferation/differentiation*** | | | | |
| Mal, T-cell differentiation protein 2 | -2.63 | Ras-related and estrogen-regulated growth inhibitor-like | | -1.44 |
|  |  | MACC1, MET transcriptional regulator | | -1.43 |
|  |  | Transmembrane protease, serine 2 | | -1.41 |
| ***Cell adhesion*** | | | | |
|  |  | Cadherin 12 | | -1.32 |
| ***Immune response*** | | | | |
|  |  | Leukocyte cell derived chemotaxin 2 | | -1.37 |
|  |  | Amphiphysin | | -1.31 |

Padj < 0.01, log_2_FC ≤ -1 was considered significant; *log_2_FC, log_2_ fold change
